# Analysis and Optimization of a General Linking Matrix for JSCC Scheme Based on Double LDPC Codes

**DOI:** 10.3390/e25020382

**Published:** 2023-02-19

**Authors:** Qiwang Chen, Zhiping Xu, Huihui Wu, Guofa Cai

**Affiliations:** 1 Xiamen Key Laboratory of Mobile Multimedia Communications, College of Information Science and Engineering, Huaqiao University, Xiamen 361021, China; 2 The School of Ocean Information Engineering, Jimei University, Xiamen 361021, China; 3 The Department of Electrical and Computer Engineering, McGill University, Montreal, QC H3A0G4, Canada; 4 The School of Information Engineering, Guangdong University of Technology, Guangzhou 510006, China

**Keywords:** linking matrix, joint source-channel coding, double low-density parity-check codes

## Abstract

A key component of the joint source-channel coding (JSCC) scheme based on double low-density parity-check (D-LDPC) codes is the introduction of a linking matrix between the source LDPC code and channel LDPC code, by which the decoding information including the source redundancy and channel state information can be transferred iteratively. However, the linking matrix is a fixed one-to-one mapping, i.e., an identity matrix in a conventional D-LDPC code system, which may not take full advantage of the decoding information. Therefore, this paper introduces a general linking matrix, i.e., a non-identity linking matrix, connecting the check nodes (CNs) of the source LDPC code and the variable nodes (VNs) of the channel LDPC code. Further, the encoding and decoding algorithms of the proposed D-LDPC coding system are generalized. A joint extrinsic information transfer (JEXIT) algorithm is derived for calculating the decoding threshold of the proposed system with a general linking matrix. In addition, several general linking matrices are optimized with the aid of the JEXIT algorithm. Finally, the simulation results demonstrate the superiority of the proposed D-LDPC coding system with general linking matrices.

## 1. Introduction

Compared with classical separate source and channel coding schemes, a common objective of joint source-channel coding (JSCC) schemes is to make full use of the source and channel information in order to boost the performance of the overall system. As a promising coding structure, a JSCC scheme consisting of two low-density parity-check (LDPC) codes was proposed in [1], where an LDPC code is for source compression and the other one is for channel coding. This scheme is referred to as the double LDPC (D-LDPC) coding system. Further, various LDPC codes have been investigated for D-LDPC systems, such as protograph LDPC codes [2,3], quasi-cyclic LDPC codes [4] and spatially coupled LDPC codes [5].

In the D-LDPC coding system, a core idea is the introduction of a linking matrix setting up the connections between the check nodes (CNs) of the source LDPC code and the variable nodes (VNs) of the channel LDPC code, and we name it as the source check–channel variable (SC-CV) linking matrix. Using the SC-CV linking matrix, the joint belief propagation (BP) decoding can iteratively exchange the source redundancy and channel state information to improve the system performance. To achieve a better performance, the non-zero column vectors in the linking matrix should connect to the VNs with larger degrees in the channel LDPC code [6]. Moreover, the linking matrix can be optimized considering the degree distribution of the check nodes (CNs) of the source LDPC code [7].

In this paper, a generalized SC-CV linking matrix is introduced into the D-LDPC coding system. The conventional SC-CV linking matrix consists of a zero matrix and an identity matrix, i.e., both the row and column weights being 1, which implies a one-to-one mapping between the source coding and channel coding. However, this mapping may not take full advantage of the source redundancy and channel state information. Therefore, a more general linking matrix is introduced for the purpose of providing more connections between the CNs of the source LDPC code and the VNs of the channel LDPC. Consequently, the identity matrix in the SC-CV linking matrix will be replaced by a non-identity matrix. Note that the identity matrix [6], including its row and column switching [7], becomes a special case of the proposed general linking matrix.

### 1.1. Related Work of D-LDPC Code Systems

In recent years, a majority of the work for D-LDPC code systems has been studied in order to improve the bit error rate (BER) performance in the water-fall region and error-floor region. The former is evaluated by the channel decoding threshold and the latter is evaluated by the source decoding threshold. For DP-LDPC code systems, a joint protograph extrinsic information transfer (JPEXIT) algorithm [8] was proposed to analyze the channel decoding threshold. Based on the JPEXIT algorithm, an important structure, i.e., a degree-2 VN, was analyzed for DP-LDPC codes [9]. Several optimized channel protographs with more degree-2 VNs were proposed to lower the channel decoding threshold [10,11]. Moreover, this degree-2 VN structure can also help improve the source decoding threshold, which is analyzed by the source PEXIT (SPEXIT) algorithm [12]. In order to improve the error-floor performance, a linking matrix connecting the VNs of the source LDPC code and the CNs of the channel LDPC code, denoted as the source variable–channel check (SV-CC) linking matrix, was introduced in [6]. Further, several SV-CC linking matrices combined with source protographs are optimized based on the generalized SPEXIT algorithm [13]. Some integrated design work [14,15,16] of the joint protograph were conducted by differential evolution [17], where the cost function is the calculation of the decoding threshold. In addition, the improved sliding window decoding algorithm [18] and the joint grouping shuffled scheduling decoding algorithm [19] were proposed for the D-LDPC codes system.

### 1.2. Contribution

However, the aforementioned D-LDPC code systems are all based on the identity SC-CV linking matrix. The introduction of the non-identity linking matrix enables a more effective decoding information (e.g., source redundancy and channel state information) transfer between the source and channel LDPC codes.

The novelty and contributions of this paper can be summarized as follows.

(1) A general SC-CV linking matrix is introduced into the D-LDPC code system and the corresponding encoding and decoding algorithms are generalized.
(2) In order to analyze the impacts of the general SC-CV linking matrix on both the channel decoding threshold and source decoding threshold, the corresponding EXIT algorithm is proposed.
(3) Given several pairs of source and channel protographs, some general linking matrices constructed by a matrix operation are optimized.

### 1.3. Paper Organization

The remainder of this paper is organized as follows. Section 2 presents the preliminaries of the conventional D-LDPC code system. In Section 3, the novel D-LDPC code system with the proposed general linking matrix is presented as well as the generalized encoding and decoding algorithms. In Section 4, the evaluation of the source and channel decoding thresholds is described based on the JPEXIT algorithm. The structure of the proposed general linking matrix is first analyzed in Section 5, and then several general linking matrices are optimized for the given source and channel protographs pairs. The simulation results and comparisons are discussed in Section 6, and finally, Section 7 draws a conclusion of this paper.

## 2. Preliminaries on Conventional D-LDPC Coding Systems

An LDPC code can be represented by the sets {V,C,E}, respectively, indicating the set of VNs, CNs and the edges between VNs and CNs. It can also be represented by a parity-check matrix $H=\{h_{ij}\}$, where $h_{ij}=0$ or 1, indicating if there is a connection from the *j*-th VN to the *i*-th CN.

Consequently, a JSCC system based on D-LDPC codes can be represented by the following parity-check matrix $H_{J}$,

(1) $$\begin{array}{c} H_{J}=\left[\begin{array}{cc} H_{S} & H_{L1}\\ H_{L2} & H_{C} \end{array}\right]=\left[\begin{array}{cc} H_{S} & H_{L1}^{*}\;0\\ H_{L2} & H_{C} \end{array}\right], \end{array}$$

where

- $H_{S}$ (size $M_{s}\times N_{s}$) is the parity-check matrix of the source LDPC code;
- $H_{C}$ (size $M_{c}\times N_{c}$) is the parity-check matrix of the channel LDPC code;
- $H_{L1}$ of dimension $M_{s}\times N_{c}$ is the *source check–channel variable (SC-CV)* linking matrix, indicating the connections between CNs of $H_{S}$ and VNs of $H_{C}$.
- $H_{L2}$ of dimension $M_{c}\times N_{s}$ is the *source variable–channel check (SV-CC)* linking matrix, indicating the connections between VNs of $H_{S}$ and CNs of $H_{C}$.

**Remark** **1.** *The non-zero*$H_{L2}$*lowers error floors but increases channel decoding thresholds for a given code pair*$H_{S}$*and*$H_{C}$. *For simplicity, the case*$H_{L2}=0$*is considered in this paper. As all that really matters is the part*$H_{L1}^{*}$, *the linking matrix only denotes the*$H_{L1}^{*}$in the following content unless otherwise stated. Thus, the joint parity-check matrix$H_{J}$*is changed to be*

(2) $$\begin{array}{c} H_{J}=\left[\begin{array}{cc} H_{S} & H_{L1}^{*}\;0\\ 0 & H_{C} \end{array}\right]=\left[\begin{array}{cc} H_{S} & H_{I}\;0\\ 0 & H_{C} \end{array}\right], \end{array}$$

In the conventional D-LDPC coding system, $H_{L1}$ consists of an identity matrix, i.e., $H_{L1}^{*}=H_{I}$ (size $M_{s}\times M_{s}$), and an all zero matrix. The identity matrix $H_{I}$ specifies the one-to-one connection between the CNs of $H_{S}$ and the VNs of $H_{C}$. A generator matrix $G_{C}$ with a systematic form $\left[I\;{\left(P_{C}\right)}^{T}\right]$ is obtained from $H_{C}$ by Gaussian elimination.

### 2.1. Encoding

For an independent and identically distributed (i.i.d.) Bernoulli ($\xi_{1}<0.5$) source, an original source sequence s ($s\in {\left\{0,1\right\}}^{1\times N_{s}}$) is encoded into codeword b by a parity-check matrix $H_{sc}^{T}$, i.e.,

(3) $$b=s{\left(H_{S}\right)}^{T},$$

where b of dimension $1\times M_{s}$ denotes the compressed source bit sequence, and ${\left(\cdot \right)}^{T}$ represents matrix transpose operation. Next, the compressed bits b is encoded by the matrix $G_{C}$, i.e.,

(4) $$c=bG_{C}=b\left[I\;{\left(P_{C}\right)}^{T}\right]=\left[b,b{\left(P_{C}\right)}^{T}\right]=\left[b,p\right],$$

where p is a parity bit sequence p (size $1\times M_{c}$). Some bits in [bp] will be punctured if a punctured LDPC code $H_{C}$ is utilized. The left bits in [bp] are modulated by binary phase-shift keying (BPSK) and sent over an additive white Gaussian noise (AWGN) channel with noise following Gaussian distribution $N(0,\sigma^{2})$, where $2\sigma^{2}=1/\left(R\times E_{b}/N_{0}\right)$ ($E_{b}$ is the average energy of each channel bit and $N_{0}$ is the average energy of channel noise). The overall channel coding rate is expressed as

(5) $$\begin{array}{c} R=\frac{N_{c}-M_{c}}{N_{c}-N_{p}}, \end{array}$$

where $N_{p}$ is the length of punctured bits.

### 2.2. Decoding

Let us first define $Z_{S}=\left[Z_{S}^{j}\right]$ ($j=1,\cdots ,N_{s}$) and $Z_{C}=\left[Z_{C}^{j}\right]$ ($j=N_{s}+1,\cdots ,N_{s}+N_{c}$). At the receiver, the corresponding initial log-likelihood ratio (LLR) of c is calculated by $Z_{C}=2y/\sigma^{2}$ for the transmitted part and 0 for the punctured part, where y is the received signal. We assume that the source statistics are known by the receiver (the source statistics can also be estimated during decoding procedure), and the LLR of sequence s is evaluated by $Z_{S}=\log\frac{1-\xi_{1}}{\xi_{1}}$. Note that the parity-check matrix $H_{J}$ in (2) can be regarded as a single LDPC code, by globally viewing the CNs and VNs of each component matrix. Accordingly, the joint decoding algorithm based on belief propagation (BP) can be performed on a single $H_{J}$, as described below.

**Figure 1:**
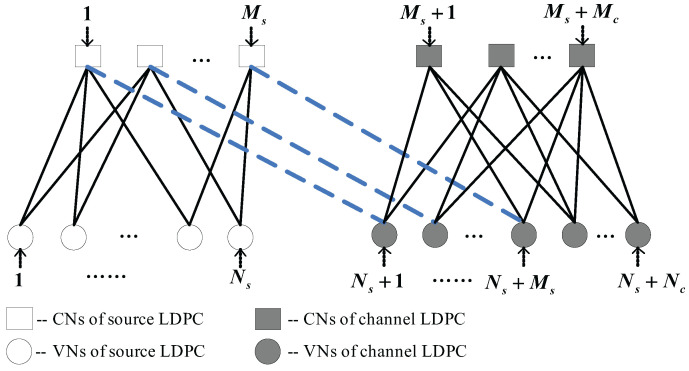
The Tanner graph of the proposed D-LDPC code system with an identity linking matrix.

Let $p_{k}=\left[p_{k}^{i,j}\right]$ (respectively, $q_{k}=\left[q_{k}^{i,j}\right]$) be the information passed from the *j*-th VN (respectively, *i*-th CN) to *i*-th CN (respectively, *j*-th VN) at the *k*-th iteration. As shown in Figure 1, the corresponding iterative decoding procedure is described as follows.

1. The information from the VNs to CNs is calculated for $j=1,2,\cdots ,N_{s}$,
  (6) $$p_{k}^{i,j}=Z_{S}^{j}+\sum_{i^{\prime }\neq i}q_{k-1}^{i^{\prime },j}$$
  and for $j=N_{s}+1,\cdots ,N_{s}+N_{c}$,
  (7) $$p_{k}^{i,j}=Z_{C}^{j}+\sum_{i^{\prime }\neq i}q_{k-1}^{i^{\prime },j}.$$
2. The information from the CNs to VNs is calculated for $i=1,2,\cdots ,M_{s}+M_{c}$, i.e.,
  (8) $$\tan h\left(\frac{q_{k}^{i,j}}{2}\right)=\prod_{j^{\prime }\neq j}\tan h\left(\frac{p_{k-1}^{i^{\prime },j}}{2}\right),$$
  where tanh(·) is the hyperbolic tangent function.
3. At the end of the *k*-th iteration, the LLRs of the estimated bits $\hat{s}=\left[{\hat{s}}_{j}\right]$ and $\hat{c}=\left[{\hat{c}}_{j}\right]$ are calculated for $j=1,2,\cdots ,N_{s}$
  (9) $$LLR{\left(s_{j}\right)}_{k}=Z_{S}^{j}+\sum_{j}q_{k}^{i,j}$$
  and for $j=N_{s}+1,\cdots ,N_{s}+N_{c}$,
  (10) $$LLR{\left(c_{j}\right)}_{k}=Z_{C}^{j}+\sum_{j}q_{k}^{i,j}.$$
4. ${\hat{c}}_{j}$(${\hat{s}}_{j}$) can be estimated by
  (11) $${\hat{c}}_{j}\left({\hat{s}}_{j}\right)=\left\{\begin{array}{cc} 0, & LLR(c_{j}\left(s_{j}\right))>0\\ 1, & \mathrm{otherwise} \end{array}\right..$$
5. If
  (12) $$\hat{c}{\left(H_{C}\right)}^{T}=0\;\mathrm{and}\;\hat{s}{\left(H_{S}\right)}^{T}=0$$
  or *k* reaches the predetermined total number *K* of iterations, the decoding will stop and turn to Step 6; otherwise, the procedure goes back to Step 1 and starts the next iteration.
6. The estimated information sequence $\hat{s}$ is obtained.

## 3. The Proposed D-LDPC Code System with a General Linking Matrix

Different from the separated source LDPC coding and channel LDPC coding systems, the conventional D-LDPC code system sets up its connections via $H_{L1}^{*}$, which is a simple identity matrix $H_{I}$ from the perspective of encoding. As for the functionality of $H_{I}$ from a decoding perspective, the information from channel decoding can be passed to source decoding, and vice versa, which allows to exchange the source redundancy and channel state information and accelerate the decoding process.

However, the one-to-one connection defined by $H_{I}$ may not make full use of this decoding information. Thus, **a general linking matrix**, i.e., non-identity matrix $H_{L1}^{*}$, denoted as $H_{P}$, will be introduced. The generalized encoding and decoding algorithm with a general linking matrix will be described as follows.

### 3.1. Generalized Encoding

The source coding procedure can be re-written as follows. A generated matrix with a systematic form $G_{S}=\left[I\;{\left(P_{S}\right)}^{T}\right]$ should first be obtained from $\left[H_{L1}^{*}\;H_{S}\right]$ by Gaussian elimination (denoted as “→”), i.e.,

(13) $$\left[H_{L1}^{*}\;H_{S}\right]=\left[H_{I}\;H_{S}\right]\to \left[I\;P_{S}\right].$$

As $H_{L1}^{*}=H_{I}$ is an identity matrix, $P_{S}$ can correspond to $H_{S}$ and $G_{S}=\left[I\;{\left(H_{S}\right)}^{T}\right]$.

Thus, in order to obtain the compressed bits b, the encoding can be calculated by

(14) $$\left[s,b\right]=sG_{S}=s\left[I\;{\left(H_{S}\right)}^{T}\right],$$

where the calculation of b is the (3).

When $H_{L1}^{*}=H_{P}$, the $\left[H_{P}\;H_{S}\right]$ should be first transformed into the systematic form, i.e.,

(15) $$\left[H_{P}\;H_{S}\right]\to \left[I\;P_{S}^{new}\right].$$

The corresponding generated matrix $G_{S}=\left[{\left(P_{S}^{new}\right)}^{T}\;I\right]$ and

(16) $$\begin{array}{c} \left[s,b\right]{\left[\begin{array}{c} H_{S}\\ H_{P} \end{array}\right]}^{T}=0. \end{array}$$

Taking the channel coding procedure (4) into consideration, the total encoding algorithm can be generalized by

(17) $$\begin{array}{cc} u & =sG_{S}G_{C}\\ \; & =s\left[I\;{\left(P_{S}^{new}\right)}^{T}\right]\left[I\;{\left(P_{C}\right)}^{T}\right]\\ \; & =\left[s,s{\left(P_{S}^{new}\right)}^{T}\right]\left[I\;{\left(P_{C}\right)}^{T}\right]\\ \; & =\left[s,b\right][I\;{\left(P_{C}\right)}^{T}]\\ \; & =\left[s,b,\left[s,b\right]{\left(P_{C}\right)}^{T}\right)]=\left[s,b,p\right]=\left[s,c\right]. \end{array}$$

### 3.2. Generalized Decoding

By doing so, the overall joint parity-check matrix $H_{J}$ can be expressed as

(18) $$\begin{array}{c} H_{J}=\left[\begin{array}{cc} H_{S} & H_{P}\;0\\ 0 & H_{C} \end{array}\right]. \end{array}$$

**Figure 2:**
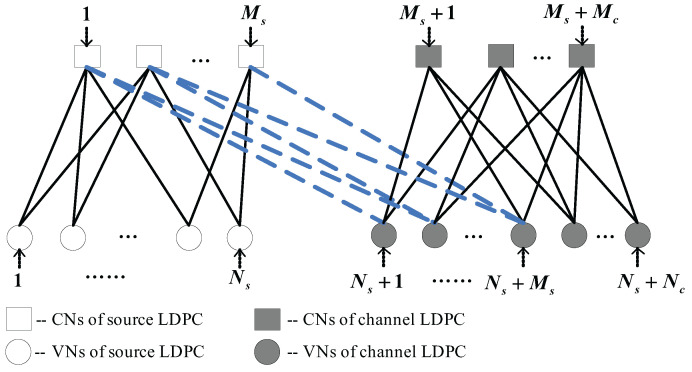
The Tanner graph of the proposed D-LDPC code system with a general linking matrix.

Considering that the vector u satisfies

(19) $$\begin{array}{cc} u{\left(H_{J}\right)}^{T} & =\left[s,b,p\right]{\left(H_{J}\right)}^{T}\\ \; & =\left[s,b,p\right]{\left[\begin{array}{cc} H_{S} & H_{P}\;0\\ 0 & H_{C} \end{array}\right]}^{T}\\ \; & =\left[s,b,p\right]\left[\begin{array}{cc} {\left(H_{S}\right)}^{T} & 0\\ \begin{array}{c} {\left(H_{P}\right)}^{T}\\ 0 \end{array} & {\left(H_{C}\right)}^{T} \end{array}\right]\\ \; & =\left[\left[s,b\right]{\left[\begin{array}{c} H_{S}\\ H_{P} \end{array}\right]}^{T},\left[b,p\right]{\left(H_{C}\right)}^{T}\right]\\ \; & =[0,0]=[0], \end{array}$$

the joint decoding procedure from (6) to (11) can be directly applied to the proposed D-LDPC coding system and the (12) should be adjusted as

(20) $$\begin{array}{c} \left[s,b\right]{\left[\begin{array}{c} H_{S}\\ H_{P} \end{array}\right]}^{T}=0\;\mathrm{and}\;\hat{c}{\left(H_{C}\right)}^{T}=0, \end{array}$$

where the joint Tanner graph of the proposed system is illustrated in Figure 2.

## 4. EXIT Analysis for the Joint Protograph with a General Linking Matrix

### 4.1. Joint Protograph

In order to illustrate the advantages of the application of the general linking matrix in the D-LDPC code system, the structured protograph LDPC codes are considered here. Different from the degree distribution representation of an irregular LDPC code, a protograph LDPC code is defined by a small protomatrix $B=\{b_{ij}\}$ ($b_{ij}$ is a non-negative integer), and a practical large parity-check matrix can be obtained by a “copy-and-permute” operation, such as the well-known progressive edge growth (PEG) algorithm [20]. The protomatrix not only clearly observes the change in code structure but also directly indicates the performance of the corresponding large parity-check matrix. Therefore, we only need to focus on the design of the small protomatrix of the corresponding large general linking matrix.

The joint protomatrix of a double protograph LDPC (DP-LDPC) code system is represented by

(21) $$\begin{array}{c} B_{J}=\left[\begin{array}{cc} B_{S} & B_{L1}^{*}\;0\\ 0 & B_{C} \end{array}\right]=\left[\begin{array}{cc} B_{S} & B_{P}\left(B_{I}\right)\;0\\ 0 & B_{C} \end{array}\right], \end{array}$$

where

- $B_{S}$ (size $m_{s}\times n_{s}$) is the protomatrix of the source protograph LDPC code;
- $B_{C}$ (size $m_{c}\times n_{c}$) is the protomatrix of the channel protograph LDPC code;
- $B_{L1}$ (size $m_{s}\times n_{c}$) is the protomatrix associated with the SC-CV linking matrix;
- $B_{P}$ of dimension $m_{p}\times n_{p}$ (note that $m_{p}=m_{s}$) is the protomatrix of the general linking matrix and $B_{I}$ is an identity protomatrix, both of them corresponding to $B_{L1}^{*}$;
- If a punctured protograph is used, then we denote by $n_{punc}$ the number of punctured VNs.

In order to obtain the corresponding parity-check matrix $H_{J}$, the PEG algorithm with a parameter $q_{1}$ is employed to remove all parallel edges at the first step. Next, the same PEG algorithm with parameter $q_{2}$ is performed again to obtain a parity-check matrix of desired sizes.

### 4.2. Joint Protograph EXIT Algorithm

In the D-LDPC coding system, two decoding thresholds should be considered, i.e., channel decoding threshold ${\left(E_{b}/N_{0}\right)}_{th}$ and source decoding threshold ${\left(\xi_{1}\right)}_{th}$. The channel decoding threshold reflects the ultimate performance in the water-fall region. On the other hand, the source decoding threshold indicates the performance of the error-floor level, and a higher ${\left(\xi_{1}\right)}_{th}$ implies a lower error-floor level.

In order to determine the channel and source decoding thresholds, the JPEXIT algorithm has to be described. Let us first define the following five types of mutual information (MI).

- $I_{e}^{v\to c}\left(i,j\right)$: the extrinsic MI from *j*-th VN to *i*-th CN;
- $I_{e}^{c\to v}\left(i,j\right)$: the extrinsic MI from *i*-th CN to *j*-th VN;
- $I_{a}^{v\to c}\left(i,j\right)$: the a prior MI from *j*-th VN to *i*-th CN;
- $I_{a}^{c\to v}\left(i,j\right)$: the a prior MI from *i*-th CN to *j*-th VN;
- $I_{app}^{v}\left(j\right)$: the MI between a posterior LLR evaluated by *j*-th VN and the corresponding bit $u_{j}$.

In addition, two functions $J_{awgn}\left(\sigma \right)$ and $J_{bsc}\left(\mu ,\xi_{1}\right)$ are defined. $J_{awgn}\left(\sigma \right)$ represents the MI between a binary bit sent over an AWGN channel and its corresponding LLR value, and it is given by

(22) $$J_{awgn}\left(\sigma \right)=1-\int_{-\infty }^{\infty }\frac{e^{-{\left(\theta -\sigma^{2}/2\right)}^{2}/\left(2\sigma^{2}\right)}}{\sqrt{2\pi \sigma^{2}}}\cdot \log_{2}\left(1+e^{-\theta }\right)d\theta .$$

Moreover, $J_{bsc}\left(\mu ,\xi_{1}\right)$ is a manipulation of the function $J_{awgn}\left(\cdot \right)$, for a binary source with an i.i.d. Bernoulli distribution ($\xi_{1}<0.5$). In other words, the equivalent channel is a binary symmetric channel with $\xi_{1}<0.5$, and it is defined as

(23) $$J_{bsc}\left(\mu ,\xi_{1}\right)=\left(1-\xi_{1}\right)I\left(V;\chi^{\left(1-\xi_{1}\right)}\right)+\xi_{1}I\left(V;\chi^{\xi_{1}}\right),$$

where I(V;χ) is the MI between the VN of the source and χ, $\chi^{\left(1-\xi_{1}\right)}∼N\left(\mu +\ln\frac{1-\xi_{1}}{\xi_{1}},2\mu \right)$ and $\chi^{\xi_{1}}∼N\left(\mu -\ln\frac{1-\xi_{1}}{\xi_{1}},2\mu \right)$.

Furthermore, the update procedure of the MI can be summarized as follows.

- **The MI update from VNs to CNs:**

For $i=1,\cdots ,m_{s}+m_{c}$ and $j=1,...,n_{s}$, if $b_{ij}\neq 0$,

(24) $$\begin{array}{c} I_{e}^{v\to c}\left(i,j\right)=J_{bsc}\left(\Upsilon_{a}^{v\to c}\left(j\right),\xi_{1}\right). \end{array}$$

For $i=1,\cdots ,m_{s}+m_{c}$ and $j=n_{s}+1,...,n_{s}+n_{c}$, if $b_{ij}\neq 0$,

(25) $$\begin{array}{c} I_{e}^{v\to c}\left(i,j\right)=J_{awgn}\left(\sqrt{\Upsilon_{a}^{v\to c}\left(j\right)+\sigma^{2}}\right), \end{array}$$

where

$$\begin{array}{cc} \Upsilon_{a}^{v\to c}\left(j\right) & =\sum_{i^{\prime }\neq i}b_{i^{\prime }j}{\left[J_{awgn}\left(I_{a}^{v\to c}\left(i^{\prime },j\right)\right)\right]}^{2}\\ \; & +\left(b_{ij}-1\right)\left[J_{awgn}\left(I_{a}^{v\to c}\left(i,j\right)\right)\right]. \end{array}$$

If $b_{ij}=0$, $I_{e}^{v\to c}\left(i,j\right)=0,j=1,\cdots ,n_{s}+n_{c}$.

For $i=1,\cdots ,m_{s}+m_{c}$ and $j=1,\cdots ,n_{s}$,

(26) $$I_{a}^{c\to v}\left(i,j\right)=I_{e}^{v\to c}\left(i,j\right).$$

- **The MI update from CNs to VNs**

For $i=1,\cdots ,m_{s}+m_{c}$ and $j=1,\cdots ,n_{s}+n_{c}$, if $b_{ij}\neq 0$,

(27) $$\begin{array}{c} I_{e}^{c\to v}\left(i,j\right)=1-J_{awgn}\left(\sqrt{\Upsilon_{a}^{c\to v}}\left(i\right)\right), \end{array}$$

where

$$\begin{array}{cc} \Upsilon_{a}^{c\to v}\left(i\right)= & \sum_{j^{\prime }\neq j}b_{ij^{\prime }}{\left[J_{awgn}^{-1}\left(1-I_{a}^{c\to v}\left(i,j^{\prime }\right)\right)\right]}^{2}\\ \; & \left(b_{ij}-1\right){\left[J_{awgn}^{-1}\left(1-I_{a}^{c\to v}\left(i,j\right)\right)\right]}^{2}. \end{array}$$

Note that $J_{awgn}^{-1}\left(I\right)$ is the inverse function of $J_{awgn}\left(\sigma \right)$, and if $b_{ij}=0$, $I_{e}^{c\to v}\left(i,j\right)=0$.

For $i=1,\cdots ,m_{s}+m_{c}$ and $j=1,\cdots ,n_{s}+n_{c}$,

(28) $$I_{a}^{v\to c}\left(i,j\right)=I_{e}^{c\to v}\left(i,j\right).$$

- **The evaluation of $I_{app}^{v}\left(j\right)$**

(29) $$\begin{array}{c} I_{app}^{v}\left(j\right)=\left\{\begin{array}{cc} J_{bsc}\left(\Upsilon_{app}^{v}\left(j\right),\xi_{1}\right), & j=1,\cdots ,n_{s}\\ J_{awgn}\left(\Upsilon_{app}^{v}\left(j\right)+\sigma^{2}\right), & j=n_{s}+1,\cdots ,n_{s}+n_{c} \end{array}\right., \end{array}$$

where

$$\begin{array}{c} \Upsilon_{app}^{v}\left(j\right)=\sum_{i}b_{ij}{\left[J_{awgn}^{-1}\left(I_{a}^{v\to c}\left(i,j\right)\right)\right]}^{2}. \end{array}$$

The above MI update procedure will be conducted iteratively until all $I_{app}^{v}\left(j\right)=1$ or the preset total number $t_{max}$ of iterations is reached. We point out that $I_{app}^{v}\left(j\right)=1$ for all $j=1,\cdots ,n_{s}+n_{c}$, implies that the decoding performance has converged.

In summary, the $I_{app}^{v}\left(j\right)$ can be viewed as a function of independent variables $B_{J}$, $\xi_{1}$, $\sigma^{2}$ and $t_{max}$, i.e.,

(30) $$I_{app}^{v}\left(j\right)=\Psi \left(B_{J},\xi_{1},\sigma^{2},t_{max}\right),$$

where $\sigma^{2}$ can be calculated from $E_{b}/N_{0}$. To this end, for a given $B_{J}$ and $\xi_{1}$, the channel decoding threshold ${\left(E_{b}/N_{0}\right)}_{th}$ is the minimum value $E_{b}/N_{0}$, making all $I_{app}^{v}\left(j\right)=1$ [10].

### 4.3. Evaluation of the Decoding Threshold

The channel decoding threshold ${\left(E_{b}/N_{0}\right)}_{th}$ indicates the performance of the water-fall regime. The conventional JPEXIT analysis only focuses on the protomatrix $B_{J}$ with an identity $B_{I}$. Because a more general linking protomatrix $B_{P}$ is introduced, the MI transfer in $B_{J}$ will be changed and thus the channel decoding threshold will be different. Note that the MI updates from (25) to (29) will still remain the same.

The source decoding threshold ${\left(\xi_{1}\right)}_{th}$ indicates the performance of the error-floor level, which is the lowest BER when $E_{b}/N_{0}$ approaches infinity. For the calculation of ${\left(\xi_{1}\right)}_{th}$ in the conventional source protograph EXIT (SPEXIT) analysis [12,13], it is assumed that the MI transfer from the channel part ($B_{C}$) to the source part ($B_{S}$) is full. Here, we set the $I_{a}^{c\to v}\left(i,j\right)=1$ for $j=n_{s}+1,\cdots n_{s}+n_{c}$ to make the information from the channel be fully correct. Thus, the source decoding threshold will not be affected by the structure of $B_{P}$ as well as $B_{C}$ and is decided by the structure of the source protomatrix $B_{S}$.

In all, introducing the general linking matrix will only affect the channel decoding threshold of the DP-LDPC coding system.

## 5. Design of General Linking Matrices

### 5.1. Structure of the General Linking Protomatrix

In order to efficiently optimize the general linking matrix, we should first discuss its structure. $H_{P}$ should be a non-singular matrix in order to keep the matrix rank of $P_{S}^{new}$ the same as that of $P_{S}$, which also keeps the length of the compressed bit b.

Therefore, the general linking protomatrix $B_{P}$ is also a non-singular matrix. From the matrix theory, the elementary row transformation is defined by three kinds of matrix operations as follows:

1. Multiply a non-zero value to one row of the matrix;
2. Add a multiple of one row in the matrix to another row;
3. Change the position of two rows in the matrix.

The elementary column transformation can also be defined via replacing the “row” with “column” in the definition above. Motivated by this, a non-singular square protomatrix (e.g., $B_{P}$) can be obtained by performing a series of elementary operations on an identity matrix (e.g., $B_{I}$). It is worth pointing out that if only operation-3 is performed for an identity matrix $B_{I}$, it corresponds to the optimization of the connections between the VNs of the source protograph and the CNs of the channel protograph [7].

**Figure 3:**
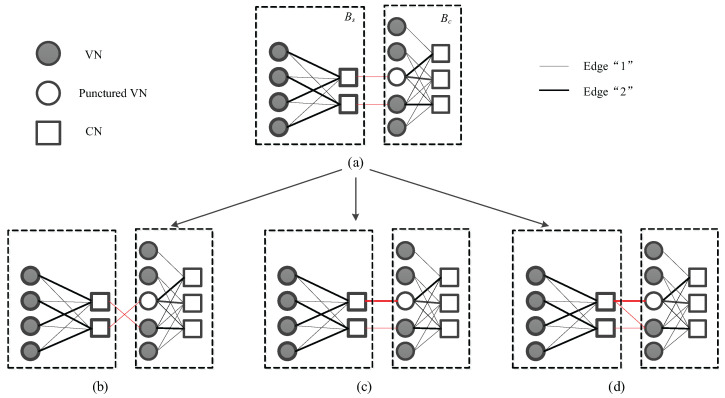
The Tanner graph (**a**) $B_{J1}^{ex1}$, (**b**) $B_{J1}^{ex2}$, (**c**) $B_{J1}^{ex3}$ and (**d**) $B_{J1}^{ex4}$ of DP-LDPC coding system with general linking protomatrix, where the red lines represent the edges in general linking protomatrix and the thickness of the line represent the edges.

In order to further illustrate the structures of the general linking protomatrix, we take the following joint protomatrix as an example, i.e.,

(31) $$\begin{array}{c} B_{J1}^{ex1}=\left[\begin{array}{ccccccccc} 2 & 1 & 2 & 1 & 0 & 0 & 0 & 1 & 0\\ 1 & 2 & 1 & 2 & 0 & 0 & 0 & 0 & 1\\ 0 & 0 & 0 & 0 & 1 & 0 & 0 & 2 & 1\\ 0 & 0 & 0 & 0 & 0 & 1 & 1 & 2 & 1\\ 0 & 0 & 0 & 0 & 0 & 1 & 1 & 1 & 2 \end{array}\right], \end{array}$$

where the linking part is a 2×2 identity matrix. The joint protograph is shown in Figure 3a. If operation-3 of the elementary transformation is performed, the joint protograph could be changed to Figure 3b, which is denoted by $B_{J1}^{ex2}$. Similarly, the protomatrix $B_{J1}^{ex3}$ shown in Figure 3c could be obtained by the operation-1, and $B_{J1}^{ex4}$ demonstrated in Figure 3d could be obtained by the combination of the operation-1 and operation-2. The protomatrix $B_{J1}^{ex4}$ is given by

(32) $$\begin{array}{c} B_{J1}^{ex4}=\left[\begin{array}{ccccccccc} 2 & 1 & 2 & 1 & 0 & 0 & 0 & 2 & 0\\ 1 & 2 & 1 & 2 & 0 & 0 & 0 & 1 & 1\\ 0 & 0 & 0 & 0 & 1 & 0 & 0 & 2 & 1\\ 0 & 0 & 0 & 0 & 0 & 1 & 1 & 2 & 1\\ 0 & 0 & 0 & 0 & 0 & 1 & 1 & 1 & 2 \end{array}\right]. \end{array}$$

### 5.2. Optimization Method

As mentioned above, the protomatrix $B_{P}$ can be constructed from the elementary matrix operations, which also determines the complexity (the row or column weight of the matrix can measure the complexity). In other words, if a set Θ consisting of all possible element values is given, the complexity of designing $B_{P}$ is determined. The complexity of the optimization also relies on the size of protomatrix, i.e., $m_{p}$. If the general linking protomatrix is optimized directly, we should design a specific structure and simultaneously optimize the connections between the CNs of source protomatrix and the VNs of channel protomatrix. In [11], it is indicated that $B_{P}$ should connect to the VNs with the largest degree in the $B_{C}$ for the optimal channel decoding threshold. Therefore, the procedures of optimizing general linking protomatrix can be summarized in three steps:

1. Select an appropriate Θ and $m_{p}$;
2. Determine the connecting VNs of the channel protomatrix by employing the identity protomatrix $B_{I}$;
3. Design the specific structure of $B_{P}$ (including the connecting CNs of the source protomatrix) based on the matrix operations of elementary row/column transformation.

### 5.3. Optimization Examples

The main tested parameters in the optimization are shown in Table 1.

**Example** **1.** *We start from a joint protomatrix $B_{J2}^{conv}$ optimized in [9], i.e.,*

(33) $$\begin{array}{c} B_{J2}^{conv}=\left[\begin{array}{c} \begin{array}{ccccccccccccc} 3 & 1 & 3 & 1 & 1 & 1 & 1 & 3 & 0 & 0 & 0 & 1 & 0\\ 1 & 3 & 1 & 3 & 1 & 2 & 2 & 1 & 0 & 0 & 0 & 0 & 1\\ 0 & 0 & 0 & 0 & 0 & 0 & 0 & 0 & 1 & 0 & 0 & 2 & 0\\ 0 & 0 & 0 & 0 & 0 & 0 & 0 & 0 & 0 & 1 & 1 & 3 & 1\\ 0 & 0 & 0 & 0 & 0 & 0 & 0 & 0 & 0 & 1 & 1 & 3 & 1 \end{array} \end{array}\right], \end{array}$$

*where $B_{S}$ is the R4JA code, $B_{C}$ is the re-designed IAR4JA code [9] and $B_{P}$ is assumed to connect the VNs of ${\left[2\;3\;3\right]}^{T}$ and ${\left[0\;1\;1\right]}^{T}$ in the $B_{C}$. The source decoding threshold shown in Table 2 is 0.028 and the channel decoding threshold at $\xi_{1}=0.01$ and 0.02 is −3.441 dB and −1.357 dB, respectively. For moderate searching complexity, the *Θ* here is set to be {0,1,2,3}. All possible $B_{P}$ are analyzed by calculating channel and source decoding thresholds. Several representative $B_{P}$ are considered and their corresponding joint protomatrices are given in Table 2, where $B_{J2}^{opt1}$ has the smallest ${\left(E_{b}/N_{0}\right)}_{th}$ and $B_{J2}^{opt-2}$ is a counterpart example. All of them have the same $B_{S}$ and $B_{C}$.*

By comparing $B_{J2}^{conv}$, $B_{J2}^{opt1}$ and $B_{J2}^{opt2}$, it is found that the source decoding threshold has little difference, but the channel decoding threshold of $B_{J2}^{opt1}$ is 0.582 dB, which is 2.084 dB lower than that of $B_{J2}^{conv}$ and $B_{J2}^{opt2}$ for source statistic $\xi_{1}=0.01$. This difference is 0.321 dB, which is 1.027 dB for source statistic $\xi_{1}=0.02$.

**Example** **2.** *Another joint protomatrix with a larger size at source statistic $\xi_{1}=0.04$ is taken as an example. The joint protomatrix includes a rate-1/2 source protograph and a rate-1/2 channel protograph given in [12]. In addition, we select Θ={0,1,2} and $m_{p}=4$. According to step-(2), the $B_{J-0.04}^{opt1}$ is given by*

(34) $$\begin{array}{c} B_{J-0.04}^{opt1}=\left[\begin{array}{cccccccccccccccc} 3 & 2 & 1 & 1 & 0 & 1 & 0 & 0 & 0 & 0 & 0 & 0 & 1 & 0 & 0 & 0\\ 2 & 3 & 1 & 0 & 1 & 0 & 1 & 0 & 0 & 0 & 0 & 0 & 0 & 1 & 0 & 0\\ 3 & 3 & 0 & 0 & 0 & 0 & 0 & 1 & 0 & 0 & 0 & 0 & 0 & 0 & 1 & 0\\ 2 & 0 & 1 & 2 & 2 & 1 & 1 & 1 & 0 & 0 & 0 & 0 & 0 & 0 & 0 & 1\\ 0 & 0 & 0 & 0 & 0 & 0 & 0 & 0 & 1 & 0 & 0 & 0 & 2 & 2 & 1 & 3\\ 0 & 0 & 0 & 0 & 0 & 0 & 0 & 0 & 0 & 1 & 0 & 0 & 3 & 0 & 0 & 3\\ 0 & 0 & 0 & 0 & 0 & 0 & 0 & 0 & 0 & 0 & 1 & 1 & 2 & 1 & 0 & 3\\ 0 & 0 & 0 & 0 & 0 & 0 & 0 & 0 & 1 & 1 & 1 & 2 & 2 & 0 & 2 & 2 \end{array}\right], \end{array}$$

*and it has a channel decoding threshold of −1.666 dB. Next, the specific structure of general linking protomatrix is optimized as*

(35) $$\begin{array}{c} B_{P-0.04}^{opt2}=\left[\begin{array}{cccc} 0 & 0 & 0 & 1\\ 0 & 1 & 0 & 2\\ 1 & 0 & 0 & 1\\ 0 & 0 & 1 & 2 \end{array}\right]. \end{array}$$

The corresponding joint protograph is denoted by $B_{J-0.04}^{opt2}$, which has a channel decoding threshold of −2.605 dB.

In the case of $\xi_{1}=0.06$, the optimized general linking protomatrix $B_{I-0.06}^{opt1}$ is the same as that in the case of $\xi_{1}=0.04$, and we denote by $B_{J-0.06}^{opt1}$ the corresponding protomatrix, with ${\left(E_{b}/N_{0}\right)}_{th}=-0.481$ dB. The optimized general linking protomatrix $B_{P-0.06}^{opt2}$ is given by

(36) $$\begin{array}{c} B_{P-0.06}^{opt2}=\left[\begin{array}{cccc} 0 & 1 & 0 & 1\\ 1 & 0 & 0 & 0\\ 0 & 0 & 0 & 1\\ 0 & 0 & 1 & 1 \end{array}\right], \end{array}$$

and the corresponding joint protograph is denoted by $B_{J-0.06}^{opt2}$, which has a channel decoding threshold of −1.149 dB. It is worth mentioning that the optimal $B_{P}$ is different for the case of $\xi_{1}=0.04$ and $\xi_{1}=0.06$, although they have the same $B_{C}$ and $B_{S}$.

By observing the optimized general linking matrix, it is found that the VN with the largest degree is located in the same column as the VN with the largest degree of the channel protomatrix. Although this is not derived by mathematical proof, such experience can reduce the search complexity of the optimization.

## 6. Simulation and Comparison Results

The BER simulation is carried out to verify the analysis of the JPEXIT algorithm. The BER simulation is performed over AWGN channels and the maximum iteration number of the joint BP decoding algorithm is set to 100.

### 6.1. The BER Simulation of Example 1

The effect of introducing the general linking protomatrix $B_{P}$ for the water-fall region and error-floor region is analyzed in this subsection. Firstly, the two-step PEG algorithm mentioned in Section 4 with parameters $q_{1}=4$ and $q_{2}=100$ is performed for $B_{J2}^{opt2}$, $B_{J2}^{opt1}$ and $B_{J2}^{conv}$, and the corresponding parity-check matrices are $H_{J2}^{opt2}$, $H_{J2}^{opt1}$ and $H_{J2}^{conv}$, respectively. Consequently, the code length is N=3200. The channel coderate *R* is 1/2.

**Figure 4:**
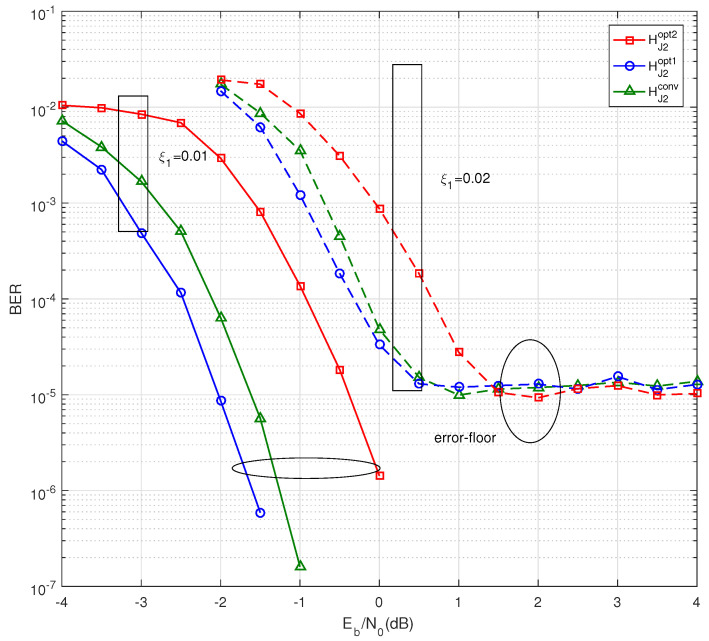
BER performance of $H_{J2}^{opt2}$, $H_{J2}^{opt1}$ and $H_{J2}^{conv}$ at source statistic $\xi_{1}=0.01$ and 0.02, respectively.

Figure 4 shows the BER performance when $\xi_{1}=0.01$ and $\xi_{1}=0.02$. For the case of $\xi_{1}=0.01$, the $H_{J2}^{opt1}$ outperforms $H_{J2}^{opt2}$ and $H_{J2}^{conv}$ by 1.57 dB and 0.37 dB at BER = $2\times 10^{-6}$, respectively. In the case of $\xi_{1}=0.02$, the coding gain in the water-fall region remains the same as the case of $\xi_{1}=0.01$, and they show an error floor at a BER = $1\times 10^{-5}$, which is almost the same level. All of the BER simulation is in line with the source and channel.

Therefore, it can be concluded that the introduction of the general linking matrix plays an important role in the water-fall region, but it has limited effect on the error-floor region.

### 6.2. The BER Simulation of Example 2

The superiority of optimizing the general linking matrix $H_{P}$ is further discussed in this subsection. For a target size of $N_{s}=3200$, the two-step PEG algorithm with $q_{1}=4$ and $q_{2}=50$ is performed for $B_{J-0.04}^{opt1}$, $B_{J-0.04}^{opt2}$, $B_{J-0.06}^{opt1}$ and $B_{J-0.06}^{opt2}$, and the corresponding parity-check matrices are $H_{J-0.04}^{opt1}$, $H_{J-0.04}^{opt2}$, $H_{J-0.06}^{opt1}$ and $H_{J-0.06}^{opt2}$, respectively.

**Figure 5:**
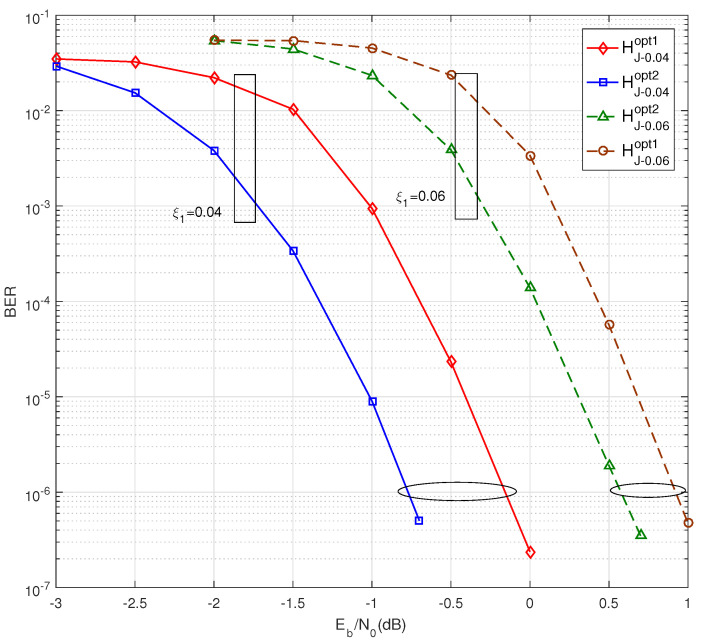
BER performance of $H_{J-0.04}^{opt1}$ and $H_{J-0.04}^{opt2}$ at source statistics $\xi_{1}=0.04$, and $H_{J-0.06}^{opt1}$ and $H_{J-0.06}^{opt2}$ at $\xi_{1}=0.06$.

Figure 5 shows the BER performance for $\xi_{1}=0.04$ and $\xi_{1}=0.06$. In the case of $\xi_{1}=0.04$, the BER performance of $H_{J-0.04}^{opt2}$ achieves a 0.62 dB coding gain over $H_{J-0.04}^{opt1}$ at a BER = $1\times 10^{-6}$. The BER performance improvement between $H_{J-0.06}^{opt1}$ and $H_{J-0.06}^{opt2}$ is only 0.36 dB for the case of $\xi_{1}=0.06$. It can be concluded that the coding gain of optimizing $H_{P}$ becomes small with an increasing source entropy for the same pair of the source protograph and channel protograph. The same phenomenon appears in Figure 4 for the comparison between $\xi_{1}=0.01$ and $\xi_{1}=0.02$.

### 6.3. Complexity Comparisons

In the BP decoding, the update in the CNs dominates the total complexity, which is represented by the average degree $\bar{d_{c}}$ of a joint protograph, shown in Table 3. The decoding complexity of the proposed joint protograph increases by 2.8%∼6.9% compared with the conventional ones, which stays in a comparable complexity level.

## 7. Conclusions

In conventional D-LDPC code systems, an identity linking matrix sets up the one-to-one connection between the source coding and channel coding, but this connection can not take full advantage of the decoding information. In this paper, a general linking matrix providing more connections is introduced into the D-LDPC code system to generalize the joint encoding procedure. Moreover, protograph LDPC codes are taken as examples for demonstrating the strengths of the proposed general linking matrices. By the aid of the JPEXIT algorithm, several general linking protomatrices to improve the channel decoding threshold are optimized. The simulation results are in line with the JPEXIT analysis, and the coding gain of the proposed DP-LDPC is 0.36–1.57 dB, which verify the superiority of the D-LDPC coding system with the proposed linking matrix.

It should be pointed out that the proposed optimization of the general linking matrix and DP-LDPC code system can also be applicable to other types of LDPC codes. The optimization of the general linking matrices over the other scenario, e.g., the joint shuffled scheduling decoding [19], the Gaussian multiple access channel [21], the non-linear modulation [22] and the non-standard coding channel [23] (e.g., optical communication [24] and an on-body channel [25]), can be studied in future work.

## Figures and Tables

**Table 1:** The main test and result parameters in the optimization.

$\xi_{1}$	probability of “1” in source bits
${\left(\xi_{1}\right)}_{th}$	the maximum value of $\xi_{1}$, i.e., source decoding threshold
$E_{b}/N_{0}$	signal-to-noise ratio
${\left(E_{b}/N_{0}\right)}_{th}$	the minimum value of $E_{b}/N_{0}$, i.e., channel decoding threshold
Error floor	the lowest BER level when $E_{b}/N_{0}\to \infty$
$B_{P}$	general linking matrix
Θ	all possible element values in protomatrix
$\bar{d_{c}}$	the average degree of CNs

**Table 2:** The channel and source decoding thresholds of several representative $B_{J2}$.

$B_{J}$	$B_{P}$	${\left(\xi_{1}\right)}_{\mathrm{th}}$	${\left(E_{b}/N_{0}\right)}_{\mathrm{th}}$	
			0.01	0.02
$B_{J2}^{conv}$	$\left[\begin{array}{cc} 1 & 0\\ 0 & 1 \end{array}\right]$	0.028	−3.441 dB	−1.357 dB
$B_{J2}^{opt1}$	$\left[\begin{array}{cc} 1 & 1\\ 0 & 2 \end{array}\right]$	0.028	−4.023 dB	−1.678 dB
$B_{J2}^{opt2}$	$\left[\begin{array}{cc} 1 & 2\\ 2 & 1 \end{array}\right]$	0.028	−1.939 dB	−0.651 dB

**Table 3:** The average degree of CNs of different joint protographs.

Conventional $B_{J}$	$\bar{d_{c}}$	Proposed $B_{J}$	$\bar{d_{c}}$	Increased Ratio
$B_{J2}^{conv}$	9.0	$B_{J2}^{opt1}$	9.4	4.4%
$B_{J-0.04}^{opt1}$	9.0	$B_{J-0.04}^{opt2}$	9.625	6.9%
$B_{J-0.06}^{opt1}$	9.0	$B_{J-0.06}^{opt2}$	9.25	2.8%

## Data Availability

Not applicable.

## Abbreviations

The following abbreviations are used in this manuscript:

AWGN	additive white Gaussian noise
APP	a posteriori probability
BER	bit error rate
BP	belief propagation
BPSK	binary phase-shift keying
CNs	check nodes
D-LDPC	double low-density parity check
EXIT	extrinsic information transfer
JEXIT	joint extrinsic information transfer
JPEXIT	joint protograph EXIT
JSCC	joint source-channel coding
LLR	log-likelihood ratio
MI	mutual information
PEG	progressive edge growth
SC-CV	source check–channel variable
SPEXIT	source protograph EXIT
SV-CC	source variable–channel check
VNs	variable nodes
